# Physical activity and the risk of frailty among community-dwelling healthy older adults: A protocol for systematic review and meta-analysis

**DOI:** 10.1097/MD.0000000000016955

**Published:** 2019-08-30

**Authors:** Bei Pan, Hongli Li, Yunhua Wang, Min Sun, Hui Cai, Jiancheng Wang

**Affiliations:** a Gansu Provincial Hospital, Lanzhou; b Hospital Management Research Center, Lanzhou University, Lanzhou; c Department of Social Medicine and Health Management, School of Public Health of Lanzhou University; d Lanzhou University Second Hospital, Lanzhou, China.

**Keywords:** community-dwelling older adults, dose-response, frailty, meta-analysis, physical activity, protocol

## Abstract

**Background:**

Frailty has become one of the biggest challenges in facilitating healthy aging. Our study aims to investigate the association between physical activity and the risk of frailty, meanwhile, conduct a dose–response to observe the relationship between physical activity volume and frailty.

**Method:**

We will systematically search six databases to identify cohort studies assessing the association between physical activity and risk of frailty. Risk of bias of individual study will be assessed using the Newcastle–Ottawa Scale. Data will be analyzed by using STATA V.12.0.

**Results:**

The results of this study will be published in a peer-reviewed journal.

**Conclusion:**

We will first investigate whether specific-physical activity reduce the risk to onset frailty in healthy older adults. However, predictable heterogeneity may exist in our study because of the inconsistent definitions of frailty.

## Abstract 2

Supplemental Digital Content is available in the text

## 1 Introduction

Population is continually growing older at an unprecedented rate throughout the world in recent centuries. According to a new report, this percentage is projected to jump to nearly 17% of the world's population by 2050 (1.6 billion).^[1]^ Although aging means prolongation of life expectancy, some problems have been raised at the same time, including the increase of social security costs and healthcare costs, decrease of labors, and how to keep older adults’ capacity to function well and live independently. Healthy aging has become an extremely challenging public health issue.

Frailty is defined as a geriatric syndrome resulted from the declines of multiple physiological systems, has become one of the biggest challenges in facilitating healthy aging.^[2,3,4]^ It is often characterized by marked vulnerability to adverse health outcomes, including falls, fractures, disability, hospitalization, long-term care, and death.^[3,5]^ The prevalence of frailty among population aged over 65 years old has been reported to range from 4% to 16% in the United States, and the prevalence is higher in patients with cancer (42%) and in nursing homes (52.3%).^[6–10]^

Exercise is believed to be the most effective management in older adults to improve quality of life and functionality.^[11]^ Regular physical activity has been estimated to reduce and postpone several adverse health outcomes in older adults with frailty, which include functional performance, cognitive impairment, sarcopenia, and depression.^[12,13]^ Recently, several systematic reviews focusing on frailty topic have been published, most of them aim to assess the effectiveness of physical activity/exercise training on adverse health consequences for older adults with frailty, or on the level or progression of frailty.^[14–18]^ For example, Puts's review focused on different interventions to prevent or reduce the level of frailty,^[16]^ Apostolo's review investigated the prevention of interventions on pre-frailty and frailty progression,^[18]^ and Jadczak's meta-analysis aimed to assess the effectiveness of interventions on physical function in frail older people.^[17]^ Obviously, none of them focused on the physical activity and the risk to onset frailty in healthy older adults. An epidemiological study showed that physical activity (walking, jogging, hiking or dancing) could reduce the risk of disability in older adults compared to sedentary.^[19]^ Cross-sectional studies showed that physical activity was associated with lower incidence of frailty.^[20,21]^ Savela's cohort study showed that high-intensity physical activity was associated with a decreased risk of both frailty and pre-frailty in initially healthy people.^[22]^

Our systematic review and meta-analysis will synthesis all available evidence, aims to investigate the association between specific types and intensity of physical activity and the risk of frailty in health older adults, and then to explore the possible dose-response relationship between physical activity volume and frailty.

## 2 Method

Our study protocol has been registered on the international prospective register of a systematic review (PROSPERO). The registration number was CRD42018090277. The systematic review protocol was planned and performed adherence to Preferred Reporting Items for Systematic review and Meta-Analysis Protocols (PRISMA-P) statement.^[23]^

### 2.1 Ethic statement

Ethics approval and patient consent are not required because this study is a meta-analysis based on the published cohort studies.

### 2.2 Search strategy

We will systematically search PubMed, Cochrane Central Register of Controlled Trials (CENTRAL), Embase, Web of science, CBM (Chinese Biological Medical Database), and CNKI (Chinese National Knowledge Infrastructure). There will be no restrictions on publication date and publication language. We have conducted a comprehensive search strategy with the help of a search specialist and will update the search in July 2019. A detailed search strategy can be found in Supplemental Digital Content (Appendix 1).

### 2.3 Inclusion and exclusion criteria

We will include studies met the following criteria:

(1) study design: longitudinal cohort studies (retrospective or prospective), a secondary analysis of randomized control trials;
(2) participants: community-dwelling older adult (aged ≥ 65);
(3) exposures: specific-types of physical activity (include leisure-time, transportation, occupational, household, sports, daily/family/community activities, and exercise), intensity and volume of physical activity;
(4) outcomes: reporting at least one variable adjusted results of odds ratios (OR), or hazard ratios (HR), or risk ratios (RR) with the 95% confidence intervals (CI);
(5) others: reporting any quantitative measures of physical activity. We will exclude abstract without detailed data, and reviews.

### 2.4 Physical activity intensity and volume

Intensity of physical activity will be classified as (1.6–2.9 metabolic equivalents (METs), <40% maximum heart rate (HRmax), or <40% maximum oxygen uptake (VO2max)); moderate (3–5.9 METs, 50–60% HR_max_, or 50–60% VO_2max_); vigorous (≥6 METs, 70–80% HR_max_, or 70–80% VO_2max_). The volume of physical activity usually was measured in inconsistent methods, including METs × minutes / hours per week, kcal of energy expenditure, hours per week, and other methods. All of those measures will be included in our study.

### 2.5 Study selection

Our search records will be imported into “Rayyan”,^[24]^ which is an online literature management platform. In order to ensure high inter-rater reliability among the reviewers, a pilot-test will be performed firstly. If the agreement among reviewers is not acceptable (Kappa <80%), we will perform multiple rounds calibration exercises. Then paired reviewers (SM, HLL, YHW, HC) will independently screen the title and abstract of all the records according to eligibility criteria. Any potentially relevant records will move to full-text screening. Any conflict will be resolved by discussing. In order to illustrate the study selection process, we will also adherence to PRISMA guidelines to form a flow diagram.^[25]^

### 2.6 Data extraction

We will use Microsoft Excel 2010 to conduct a standard data abstraction sheet to collect data of interest. Paired reviewers (SM and HLL, YHW and HC) will extract the following data: the first author, year of publication, study name, country in which study were conducted, sample size, follow up period, mean age, exposures, physical intensity (reported as MET, HR_max_, VO_2max_), physical activity volume (reported as either MET-hours/minutes per week or kcal of energy expenditure), frailty assessment scales, physical activity assessment scales, relative risk estimates (hazard ratio (HR), risk ratio (RR), and odds ratio (OR)) with the 95% CI, and variables adjusted for in the study. Any conflict will be resolved by discussing.

### 2.7 Risk of bias assessment

Risk of bias of included studies will be assessed according to the Newcastle–Ottawa Scale (NOS)^[26]^ including 3 subscales as follows:

Selection

(1) representativeness of the exposed cohort;
(2) selection of the non-exposed cohort;
(3) ascertainment of exposure;
(4) demonstration that outcome of interest was not present at start of study.

Comparability

(1) comparability of cohorts on the basis of the design or analysis
(2) adjustment of confounders in the analysis.

Outcome

(1) assessment of outcome;
(2) was follow-up long enough for outcomes to occur;
(3) adequacy of follow up of cohorts.

We will consider studies with scores of less than 4 to have a high risk of bias, those with scores of 4 to 6 a medium risk of bias, and those with scores of 7 or more a low risk of bias.^[26]^ The risk of bias assessment will be assessed by paired reviewers (BP and HLL), and conflict will be resolved by a third reviewer (JCW).

### 2.8 Meta-analysis

We will use STATA V.12.0 software (Stata Corporation, College Station, TX) to conduct our meta-analysis. We will consider unadjusted and adjusted measures of effect separately. We will first pool ratio measures of effect including HR, RR, and OR using random effects model. Then, we plan to perform a sensitivity analysis using only studies that reported time-to-event data (HR) to observe the robustness of the results.^[27]^ Dose-response analysis will be conducted to investigate the relationship between physical activity volume and frailty risk by using generalized least squares trend estimation to computing regression slopes (trends) across different physical activity levels.^[28]^ Heterogeneity of treatment effects across trials will be assessed using the *Q* test with the DerSimonian-Laird method, and quantify heterogeneity using *I*^2^ statistics. We will consider 25%, 50%, and 75% as low, moderate and high 25%, 50%, and 75% heterogeneity, respectively.^[29]^

### 2.9 Subgroup analysis

In order to examine the effect of study-level variables on the association between physical activity and frailty, we will perform a priori subgroup analysis if there are more than 2 studies for each subgroup factor.^[30]^ We will consider the following effect modified factors: age, gender, adjustment for confounding factors, risk of bias, and types of physical activity. In addition, we will classify the definition of frailty into stricter and less strict based on the reporting of included studies.

### 2.10 Publication bias

We will draw a funnel plot to identify the possibility of publication bias using STATA V.12.0 software (Stata Corporation, College Station, TX).

## Author contributions

**Conceptualization:** Bei Pan, Jiancheng Wang.

**Investigation:** Bei Pan, Hong Li Li, Yun Hua Wang, Min Sun, Hui Cai.

**Methodology:** Bei Pan, Hong Li Li.

**Project administration:** Hong Li Li, Yun Hua Wang, Min Sun.

**Writing – original draft:** Bei Pan.

**Writing – review & editing:** Bei Pan, Hong Li Li, Yun Hua Wang, Min Sun, Hui Cai, Jiancheng Wang.

## Supplementary Material

Supplemental Digital Content

## References

[1] Aging, National Institute on (2016-03-28). “World's older population grows dramatically”. National Institute on Aging. Retrieved 2019-05-05.

[2] Fried LP Tangen CM Walston J Frailty in older adults: evidence for a phenotype. J Gerontol A Biol Sci Med Sci 2001;56:M146eM156. 11253156 10.1093/gerona/56.3.m146

[3] Ferrucci L Guralnik J Studenski S Designing randomized, controlled trials aimed at preventing or delaying functional decline and disability in frail, older persons: a consensus report. J Am Geriatr Soc 2004;52:625–34. 15066083 10.1111/j.1532-5415.2004.52174.x

[4] Clegg A Young J IIiffe S Frailty in elderly people. Lancet 2013;381:752–62. 23395245 10.1016/S0140-6736(12)62167-9 PMC4098658

[5] Kojima G Frailty as a predictor of hospitalisation among community-dwelling older people: a systematic review and meta-analysis. J Epidemiol Community Health 2016;70:722e729. 26933121 10.1136/jech-2015-206978

[6] Cawthon PM Marshall LM Michael Y Frailty in older men: prevalence, progression, and relationship with mortality. J Am Geriatr Soc 2007;55:1216e1223. 17661960 10.1111/j.1532-5415.2007.01259.x

[7] Santos-Eggimann B Cuenoud P Spagnoli J Prevalence of frailty in middle-aged and older community-dwelling Europeans living in 10 countries. J Gerontol A Biol Sci Med Sci 2009;64:675e681. 19276189 10.1093/gerona/glp012 PMC2800805

[8] Handforth C Clegg A Young C The prevalence and outcomes of frailty in older cancer patients: a systematic review. Ann Oncol 2015;26:1091e1101. 25403592 10.1093/annonc/mdu540

[9] Kojima G Prevalence of frailty in nursing homes: a systematic review and meta-analysis. J Am Med Dir Assoc 2015;16:940e945. 26255709 10.1016/j.jamda.2015.06.025

[10] Song X Mitnitski A Rockwood K Prevalence and 10-year outcomes of frailty in older adults in relation to deficit accumulation. J Am Geriatr Soc 2010;58:681–7. 20345864 10.1111/j.1532-5415.2010.02764.x

[11] Jobson MD Second-generation antipsychotic medications: pharmacology, administration, and side effects. Post TW, ed. UpToDate. Waltham, MA: UpToDate Inc Available at: https://www.uptodate.com/contents/frailty#H598978339 [access date 15 Jun, 2018].

[12] Peterson MJ Giuliani C Morey MC Physical activity as a preventative factor for frailty: the health, aging, and body composition study. J Gerontol A Biol Sci Med Sci 2009;64:61–8. 19164276 10.1093/gerona/gln001 PMC2913907

[13] Landi F Abbatecola AM Provinciali M Moving against frailty: does physical activity matter? Biogerontology 2010;11:537–45. 20697813 10.1007/s10522-010-9296-1

[14] de Vries NM van Ravensberg CD Hobbelen JS Effects of physical exercise therapy on mobility, physical functioning, physical activity and quality of life in community-dwelling older adults with impaired mobility, physical disability and/or multi-morbidity: a meta-analysis. Ageing Res Rev 2012;11:136–49. 22101330 10.1016/j.arr.2011.11.002

[15] Theou O Stathokostas L Roland KP The effectiveness of exercise interventions for the management of frailty: a systematic review. J Aging Res 2011;2011:569194. 21584244 10.4061/2011/569194 PMC3092602

[16] Puts MTE Toubasi S Andrew MK Interventions to prevent or reduce the level of frailty in community-dwelling older adults: a scoping review of the literature and international policies. Age Ageing 2017;46:383–92. 28064173 10.1093/ageing/afw247 PMC5405756

[17] Jadczak AD Makwana N Luscombe-Marsh N Effectiveness of exercise interventions on physical function in community-dwelling frail older people: an umbrella review of systematic reviews. JBI Database Syst Rev Implement Rep 2018;16:752–75. 10.11124/JBISRIR-2017-003551 29521871

[18] Apóstolo J Cooke R Bobrowicz-Campos E Effectiveness of interventions to prevent pre-frailty and frailty progression in older adults: a systematic review. JBI Database Syst Rev Implement Rep 2018;16:140–232. 10.11124/JBISRIR-2017-003382 PMC5771690 29324562

[19] Wu SC Leu SY Li CY Incidence of and predictors for chronic disability in activities of daily living among older people in Taiwan. J Am Geriatr Soc Sep 1999;47:1082–6. 10.1111/j.1532-5415.1999.tb05231.x 10484250

[20] Blodgett J Theou O Kirkland S The association between sedentary behaviour, moderate–vigorous physical activity and frailty in NHANES cohorts. Maturitas 2015;80:187–91. 25542406 10.1016/j.maturitas.2014.11.010

[21] Huisingh-Scheetz M Wroblewski K Kocherginsky M Physical activity and frailty among older adults in the U.S. based on hourly accelerometry data. J Gerontol A Biol Sci Med Sci 2018;73:622–9. 29106478 10.1093/gerona/glx208 PMC5905616

[22] Savela SL Koistinen P Stenholm S Leisure-time physical activity in midlife is related to old age frailty. J Gerontol A Biol Sci Med Sci 2013;68:1433–8. 23525478 10.1093/gerona/glt029

[23] Shamseer L Moher D Clarke M Preferred reporting items for systematic review and meta-analysis protocols (PRISMA-P) 2015: elaboration and explanation. BMJ 2015;349:g7647. 10.1136/bmj.g7647 25555855

[24] Ouzzani M Hammady H Fedorowicz Z Rayyan-a web and mobile app for systematic reviews. Syst Rev 2016;5:210. 27919275 10.1186/s13643-016-0384-4 PMC5139140

[25] Moher D Liberati A Tetzlaff J Preferred reporting items for systematic reviews and meta-analyses: the PRISMA statement. Int J Surg 2010;8:336–41. 20171303 10.1016/j.ijsu.2010.02.007

[26] Wells G Shea B O’Connell D The Newcastle–Ottawa Scale (NOS) for assessing the quality of nonrandomised studies in meta-analyses. Available at: http://www.ohri.ca/programs/clinical_epidemiology/oxford.asp [access date 30 Jun 2019].

[27] Bhindi B Wallis CJD Nayan M The association between vasectomy and prostate cancer: a systematic review and meta-analysis. JAMA Intern Med 2017;177:1273–86. 28715534 10.1001/jamainternmed.2017.2791 PMC5710573

[28] Greenland S longnecker MP Methods for trend estimation from summarized dose-response data, with applications to meta-analysis. Am J Epidemiol 1992;135:1301–30. 1626547 10.1093/oxfordjournals.aje.a116237

[29] Samitz G Egger M Zwahlen M Domains of physical activity and all-cause mortality: systematic review and dose–response meta-analysis of cohort studies. Int J Epidemiol 2011;40:1382–400. 22039197 10.1093/ije/dyr112

[30] Sterne JAC Juni P Schulz KF Statistical methods for assessing the influence of study characteristics on treatment effects in “meta-epidemiological” research. Statist Med 2002;21:1513–24. 10.1002/sim.1184 12111917

