# Physical activity and the risk of frailty among community-dwelling healthy older adults: A protocol for systematic review and meta-analysis: Erratum

**DOI:** 10.1097/MD.0000000000017442

**Published:** 2019-09-27

In the article, “Physical activity and the risk of frailty among community-dwelling healthy older adults: A protocol for systematic review and meta-analysis”,^[1]^ which appears in Volume 98, Issue 35 of *Medicine*, Drs. Bei Pan and Hongli Li are co-first authors.
