# Supplementary material for: Physical activity and the risk of frailty among community-dwelling healthy older adults: A protocol for systematic review and meta-analysis
Source: Medicine (Baltimore). 2019 Aug 30;98(35):e16955. doi: 10.1097/MD.0000000000016955 (PMC6736461; doi:10.1097/MD.0000000000016955)
Supplement: Supplemental Digital Content [file medi-98-e16955-s001.pdf]

Appendix 1 Search strategies

PubMed:

Search ((((("Frailty"[Mesh]) OR "Frail Elderly"[Mesh]) OR frailty[Title/Abstract]) OR frail[Title/Abstract])) AND (("Exercise"[Mesh]) OR ((((((((((((((((((((((((((((("combined training"[Title/Abstract]) OR "combined exercise"[Title/Abstract]) OR "weight-lifting"[Title/Abstract]) OR "weight lifting"[Title/Abstract]) OR Sports[Title/Abstract]) OR walking[Title/Abstract]) OR jogging[Title/Abstract]) OR swimming[Title/Abstract]) OR running[Title/Abstract]) OR treadmill[Title/Abstract]) OR accelerometer[Title/Abstract]) OR yoga[Title/Abstract]) OR bicycling[Title/Abstract]) OR "Leisure Activities"[Mesh]) OR "tai chi"[Title/Abstract]) OR "aerobic exercise"[Title/Abstract]) OR "aerobic exercises"[Title/Abstract]) OR "aerobic training"[Title/Abstract]) OR "endurance exercise"[Title/Abstract]) OR "endurance exercises"[Title/Abstract]) OR "endurance training"[Title/Abstract]) OR "resistance exercise"[Title/Abstract]) OR "resistance exercises"[Title/Abstract]) OR "strength exercises"[Title/Abstract]) OR "strength training"[Title/Abstract]) OR exercise[Title/Abstract]) OR exercises[Title/Abstract]) OR "physical Exercise"[Title/Abstract]) OR "Physical Exercises"[Title/Abstract]) OR "Physical activity"[Title/Abstract]) OR "physical activities"[Title/Abstract]))

Cochrane Library:

#1 MeSH descriptor: [Exercise] explode all trees 20039

#2 MeSH descriptor: [Leisure Activities] explode all trees 16017

#3 "combined training":ti,ab,kw or "combined exercise":ti,ab,kw or "weight-lifting":ti,ab,kw or "weight lifting":ti,ab,kw or Sports:ti,ab,kw (Word variations have been searched) 6371

#4 walking:ti,ab,kw or jogging:ti,ab,kw or running:ti,ab,kw or swimming:ti,ab,kw or treadmill:ti,ab,kw (Word variations have been searched) 31170

#5 accelerometer:ti,ab,kw or yoga:ti,ab,kw or "tai chi":ti,ab,kw or bicycling:ti,ab,kw or "Leisure Activities":ti,ab,kw (Word variations have been searched) 8012

#6 "aerobic exercise":ti,ab,kw or "aerobic exercises":ti,ab,kw or "aerobic training":ti,ab,kw or "endurance exercise":ti,ab,kw or "endurance exercises":ti,ab,kw (Word variations have been searched) 5534

#7 "endurance training":ti,ab,kw or "resistance exercise":ti,ab,kw or "resistance exercises":ti,ab,kw or "strength exercises":ti,ab,kw or "strength training":ti,ab,kw (Word variations have been searched) 5270

#8 exercise:ti,ab,kw or exercises:ti,ab,kw or "physical Exercise":ti,ab,kw or "Physical Exercises":ti,ab,kw or "Physical activity":ti,ab,kw (Word variations have been searched) 68413

#9 "physical activities":ti,ab,kw (Word variations have been searched) 14928

#10 #1 or #2 or #3 or #4 or #5 or #6 or #7 or #8 or #9 92530

#11 MeSH descriptor: [Frail Elderly] explode all trees 672

#12 Frailty:ti,ab,kw or frail:ti,ab,kw (Word variations have been searched) 1877

#13 #11 or #12 1877

#14 #10 and #13 611

Embase:

#1 frailty:ab,kw,ti OR 'frail elderly':ab,kw,ti OR frail:ab,kw,ti

#2'combined training':ab,kw,ta OR 'combined exercise':ab,kw,ta OR 'weight-lifting':ab,kw,ta OR 'weight lifting':ab,kw,ta OR sports:ab,kw,ta OR walking:ab,kw,ta OR jogging:ab,kw,ta OR swimming:ab,kw,ta OR running:ab,kw,ta OR treadmill:ab,kw,ta OR accelerometer:ab,kw,ta OR yoga:ab,kw,ta OR bicycling:ab,kw,ta OR 'leisure activities':ab,kw,ta OR 'tai chi':ab,kw,ta OR 'aerobic exercise':ab,kw,ta OR 'aerobic exercises':ab,kw,ta OR 'aerobic training':ab,kw,ta OR 'endurance exercise':ab,kw,ta OR 'endurance exercises':ab,kw,ta OR 'endurance training':ab,kw,ta OR 'resistance exercise':ab,kw,ta OR 'resistance exercises':ab,kw,ta OR 'strength exercises':ab,kw,ta OR 'strength training':ab,kw,ta OR exercise:ab,kw,ta OR exercises:ab,kw,ta OR 'physical exercise':ab,kw,ta OR 'physical exercises':ab,kw,ta OR 'physical activity':ab,kw,ta OR 'physical activities':ab,kw,ti

#3 'weight-lifting'/exp OR 'weight lifting'/exp OR 'sports'/exp OR 'walking'/exp OR 'jogging'/exp OR 'swimming'/exp OR 'running'/exp OR 'treadmill'/exp OR 'accelerometer'/exp OR 'yoga'/exp OR 'bicycling'/exp OR 'leisure activities'/exp OR 'tai chi'/exp OR 'aerobic exercise'/exp OR 'aerobic training'/exp OR 'aerobic training' OR 'endurance exercise'/exp OR 'endurance training'/exp OR 'resistance exercise'/exp OR 'resistance exercise' OR 'strength training'/exp OR 'exercise'/exp OR 'physical exercise'/exp OR 'physical activity'/exp

#4 #2 OR #3

#5 #4 AND #1

#6 AND [embase]/lim NOT [medline]/lim
